# Supplementary material for: Purification of recombinant bacterial collagens containing structural perturbations
Source: PLoS One. 2023 May 17;18(5):e0285864. doi: 10.1371/journal.pone.0285864 (PMC10191275; doi:10.1371/journal.pone.0285864)
Supplement: S1 File — (PDF) [file pone.0285864.s001.pdf]

Supporting Information

Purification of recombinant bacterial collagens containing structural perturbations

Sonal Gahlawat[1], Vikas Nanda[2,3], David I. Shreiber[1*]

[1]Department of Biomedical Engineering, Rutgers, The State University of New Jersey, Piscataway, NJ, USA

[2]Department of Biochemistry and Molecular Biology, Robert Wood Johnson Medical School, Rutgers, The State University of New Jersey, Piscataway, NJ, USA

[3]Center for Advanced Biotechnology and Medicine, Rutgers, The State University of New Jersey, Piscataway, NJ, USA

*Corresponding author

Email: shreiber@soe.rutgers.edu

The PDF file includes Supplementary Figures 1 to 13.

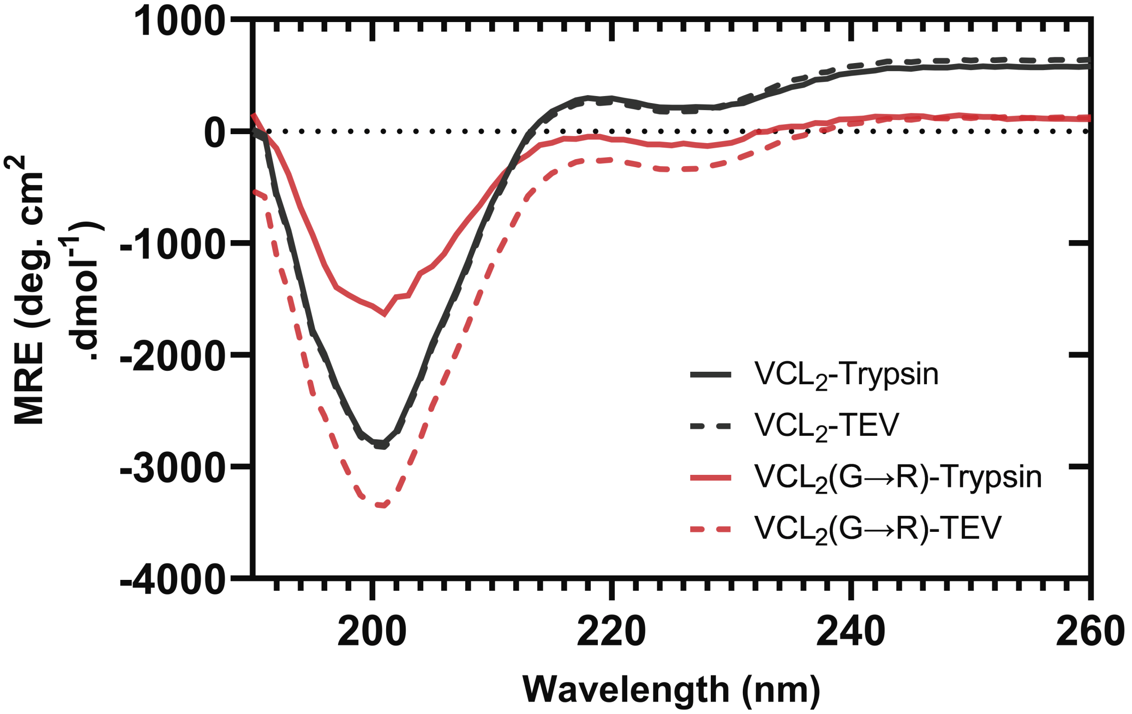

Fig S1. Circular Dichroism (CD) spectra of purified proteins: VCL_2-Trypsin, VCL_2-TEV, VCL_2(G→R)-Trypsin, and VCL_2(G→R)-TEV.

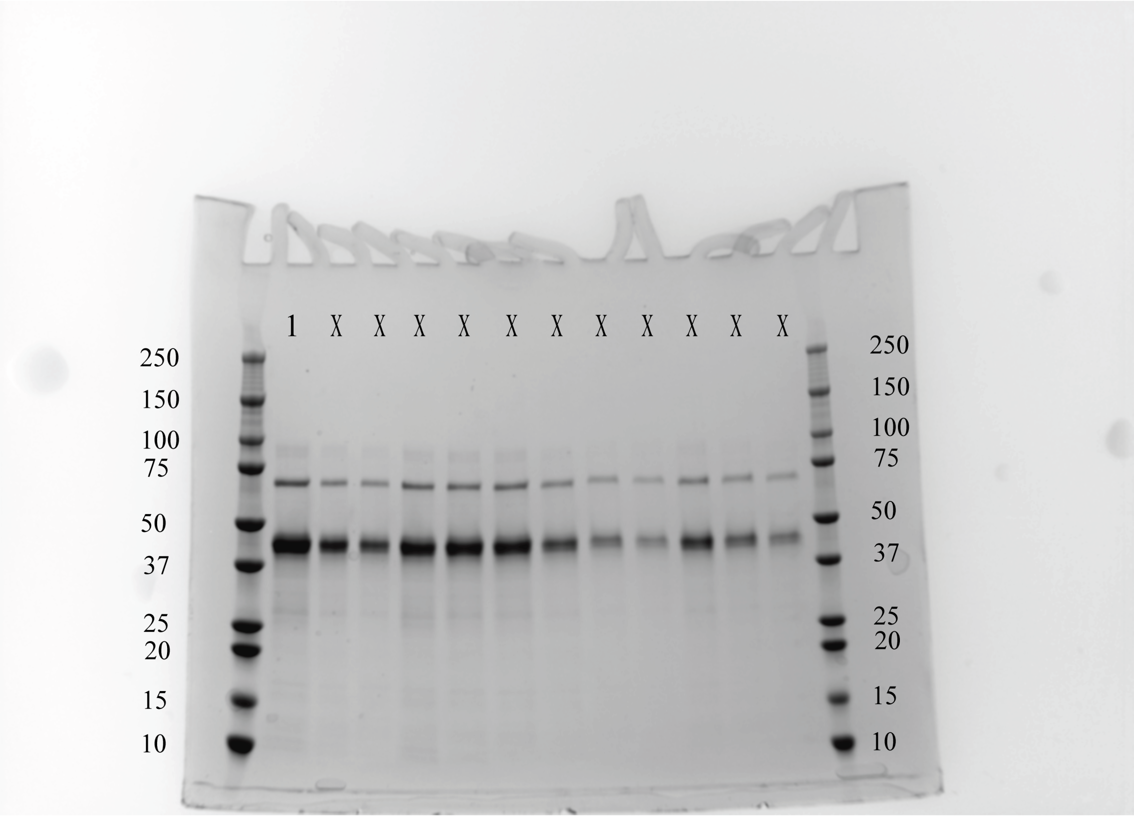

Fig S2. SDS-PAGE of purified VCL_2 in *E. coli* BL21 strain (Lane 1). Gels were stained with Coomassie blue, destained, and imaged using Gel Doc Imager. Lane 1 was used to generate Fig 3A in the manuscript. Molecular weight standards are in kDa.

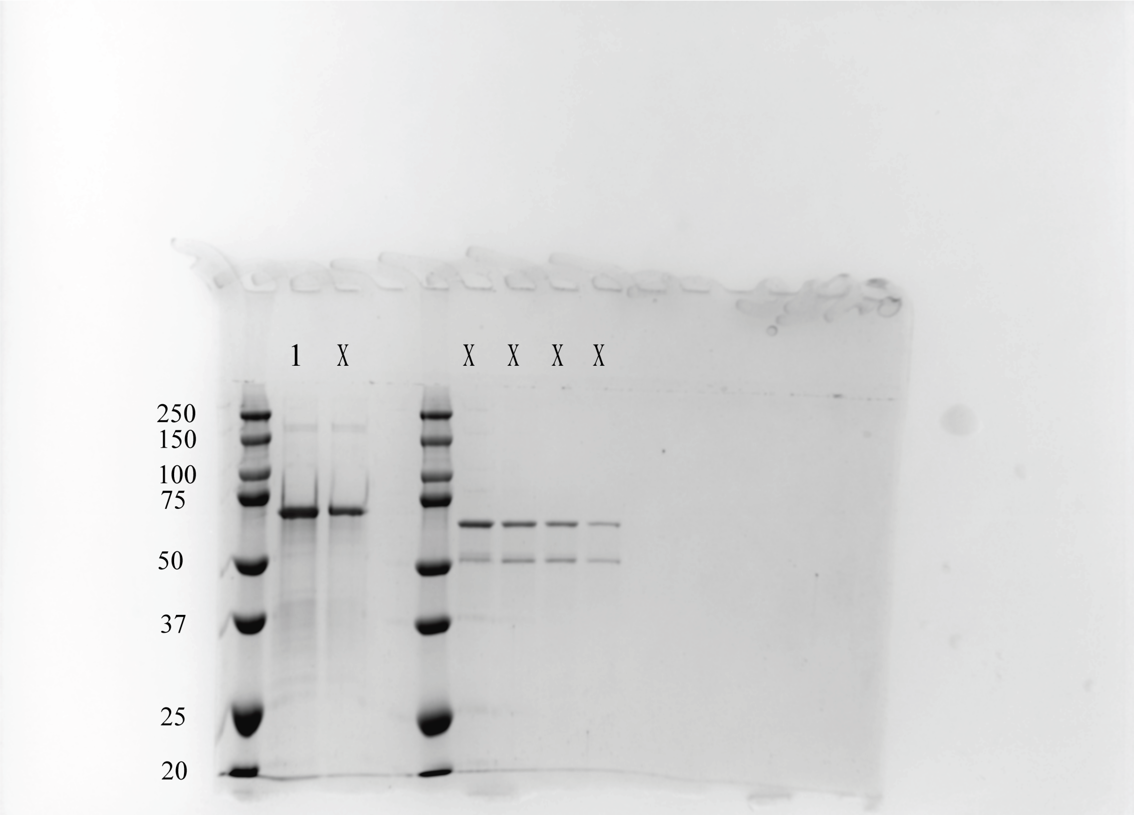

Fig S3. SDS-PAGE of purified VCL_2 in *E. coli* BL21-DE3 strain (Lane 1). Gels were stained with Coomassie blue, destained, and imaged using Gel Doc Imager. Lane 1 was used to generate Fig 3A in the manuscript. Molecular weight standards are in kDa.

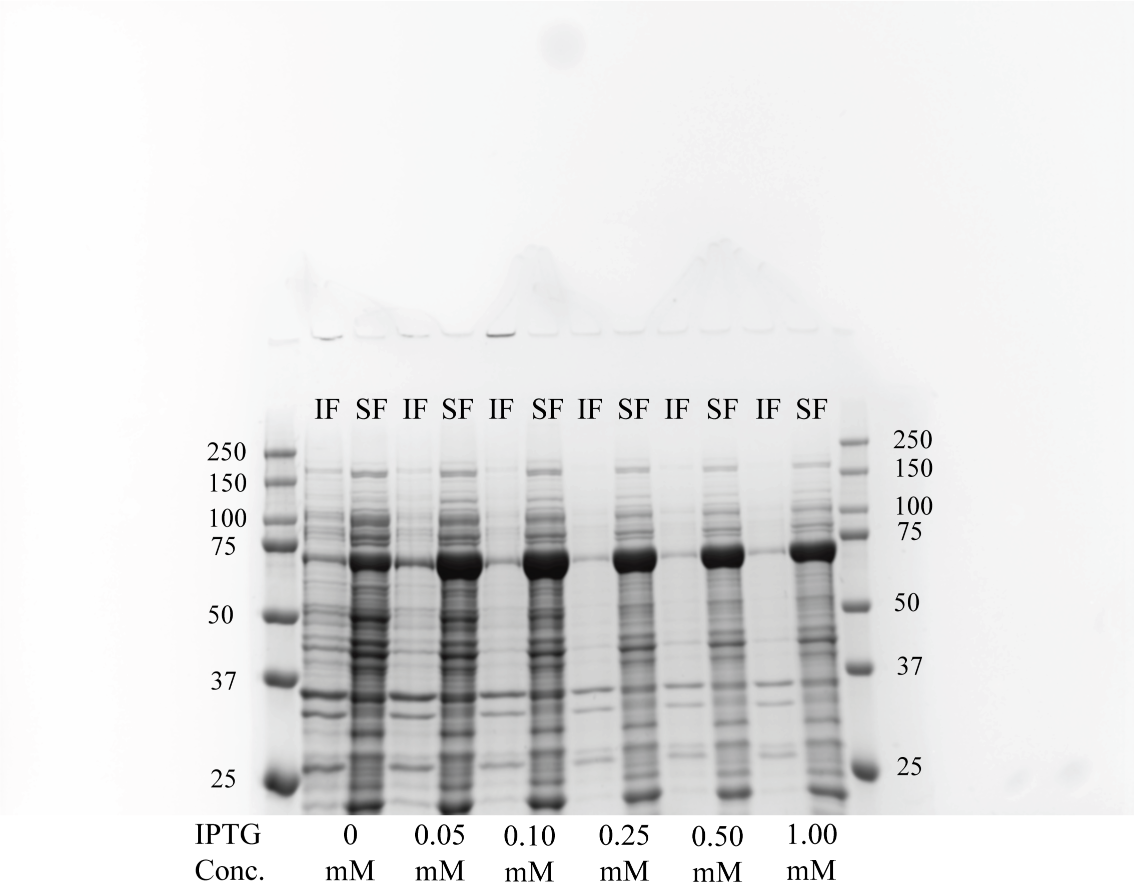

Fig S4. SDS-PAGE of insoluble (IF) and soluble fractions (SF) of VCL_2 in *E. coli* BL21-DE3 after induction with varying concentrations of IPTG. Gels were stained with Coomassie blue, destained, and imaged using Gel Doc Imager. The entire gel image was used to generate Fig 3B in the manuscript. Molecular weight standards are in kDa. Conc. = Concentration.

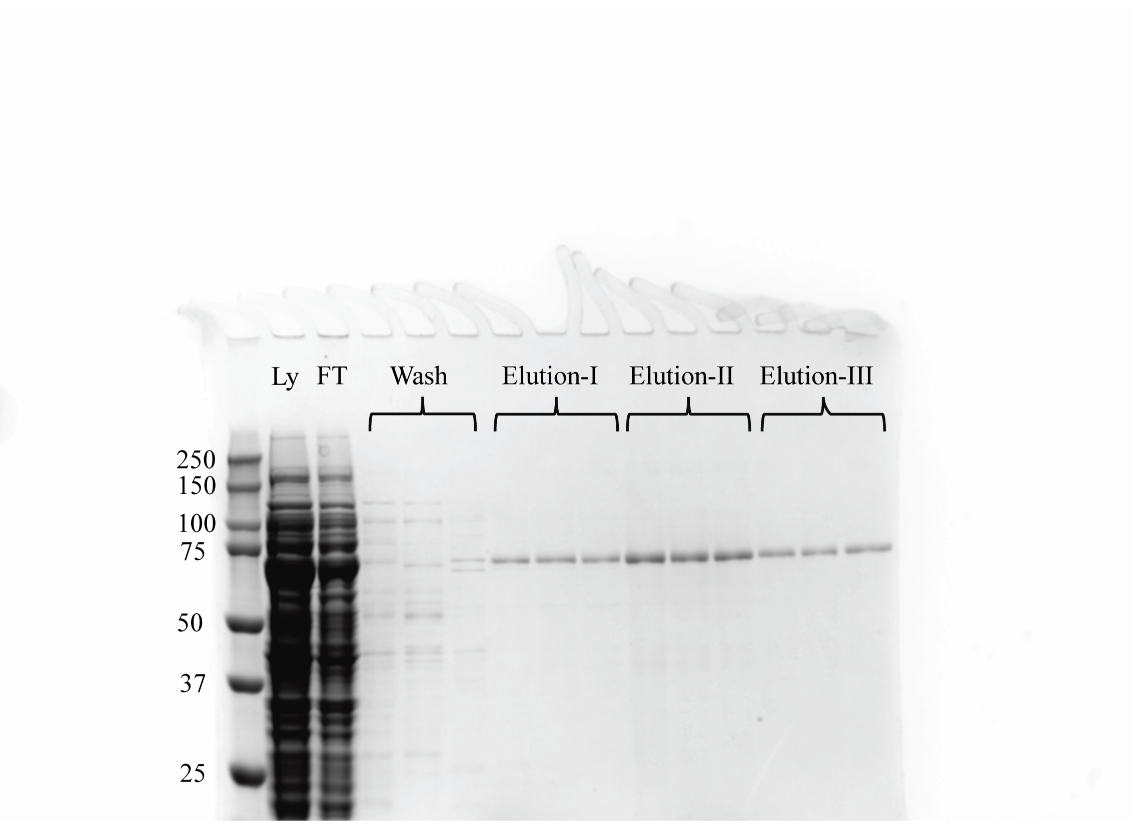

Fig S5. SDS-PAGE of the purified VCL_2-Trypsin using Ni-NTA purification. Gels were stained with Coomassie blue, destained, and imaged using Gel Doc Imager. The entire gel image was used to generate Fig 4A in the manuscript. Molecular weight standards are in kDa.

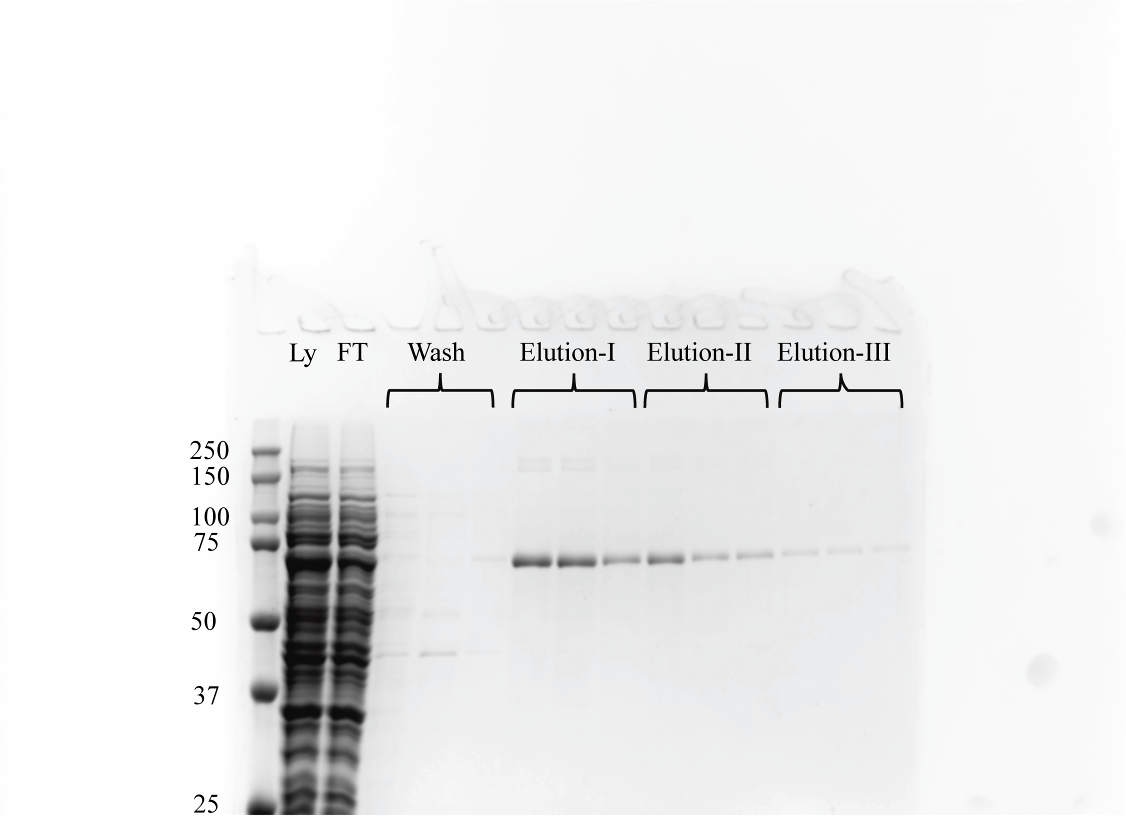

Fig S6. SDS-PAGE of the purified VCL_2-TEV using Ni-NTA purification. Gels were stained with Coomassie blue, destained, and imaged using Gel Doc Imager. The entire gel image was used to generate Fig 4B in the manuscript. Molecular weight standards are in kDa.

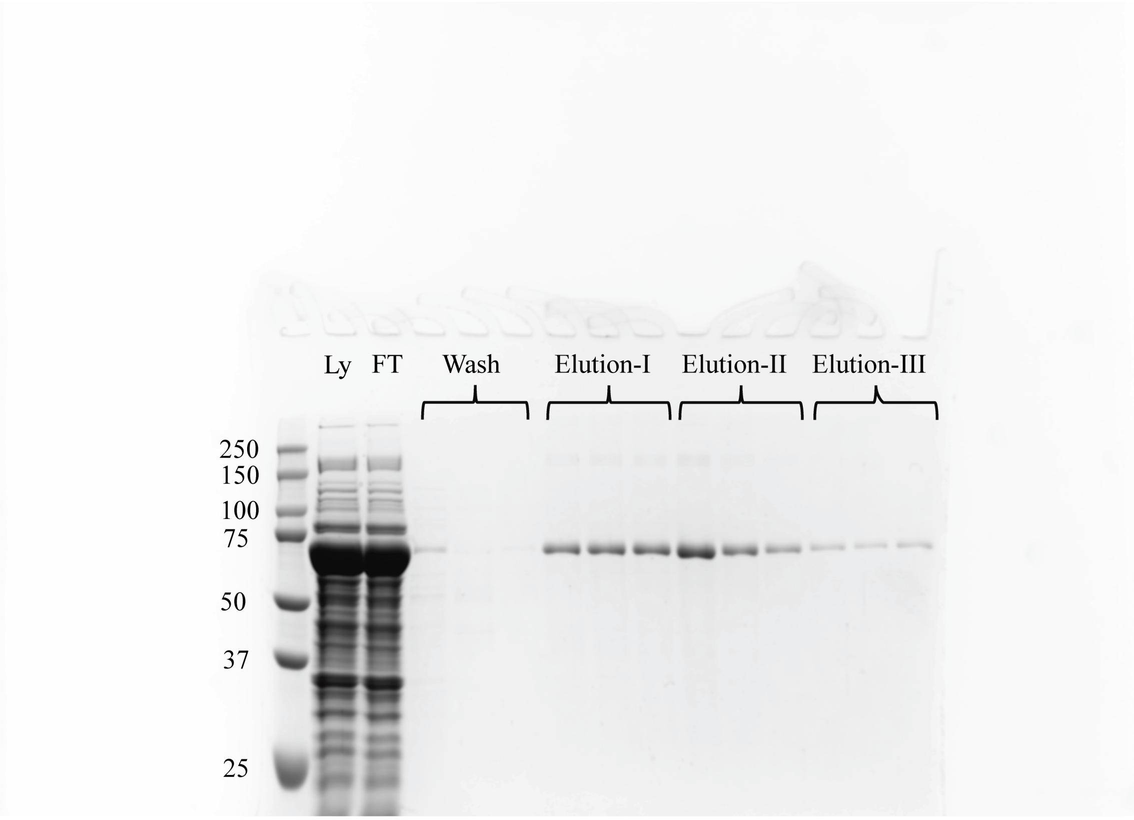

Fig S7. SDS-PAGE of the purified VCL₂(G→R)-Trypsin using Ni-NTA purification. Gels were stained with Coomassie blue, destained, and imaged using Gel Doc Imager. The entire gel image was used to generate Fig 4C in the manuscript. Molecular weight standards are in kDa.

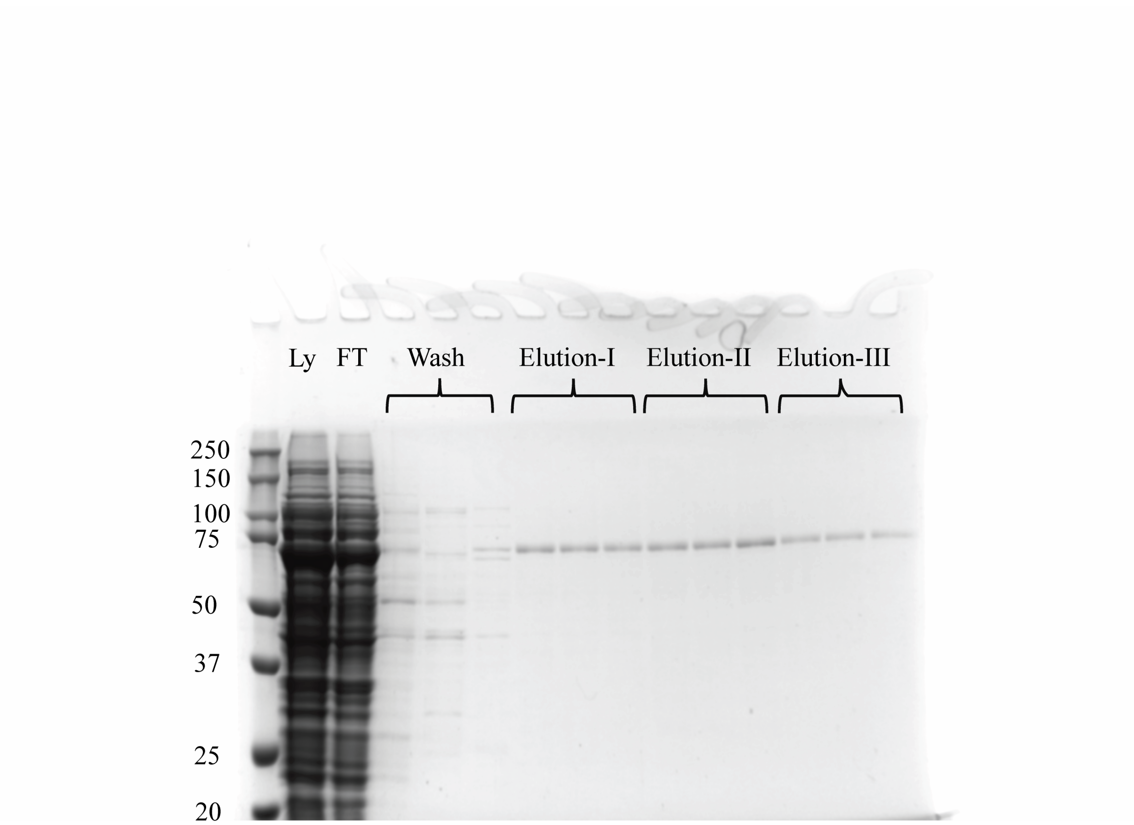

Fig S8. SDS-PAGE of the purified VCL₂(G→R)-TEV using Ni-NTA purification. Gels were stained with Coomassie blue, destained, and imaged using Gel Doc Imager. The entire gel image was used to generate Fig 4D in the manuscript. Molecular weight standards are in kDa.

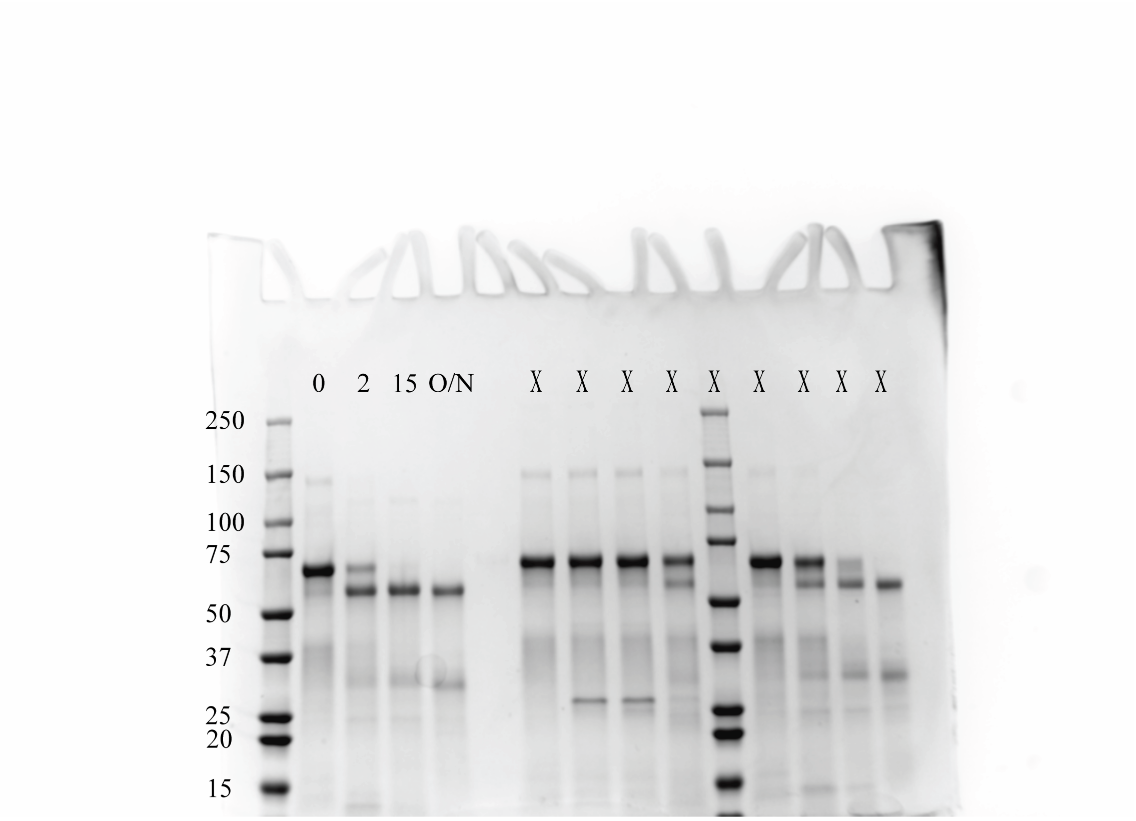

Fig S9. SDS-PAGE of enzymatic digestion of purified VCL_2-Trypsin with trypsin for different time points (0 mins, 2 mins, 15 mins, and overnight (O/N)). Gels were stained with Coomassie blue, destained, and imaged using Gel Doc Imager. The gel image was used to generate Fig 5A in the manuscript. Molecular weight standards are in kDa.

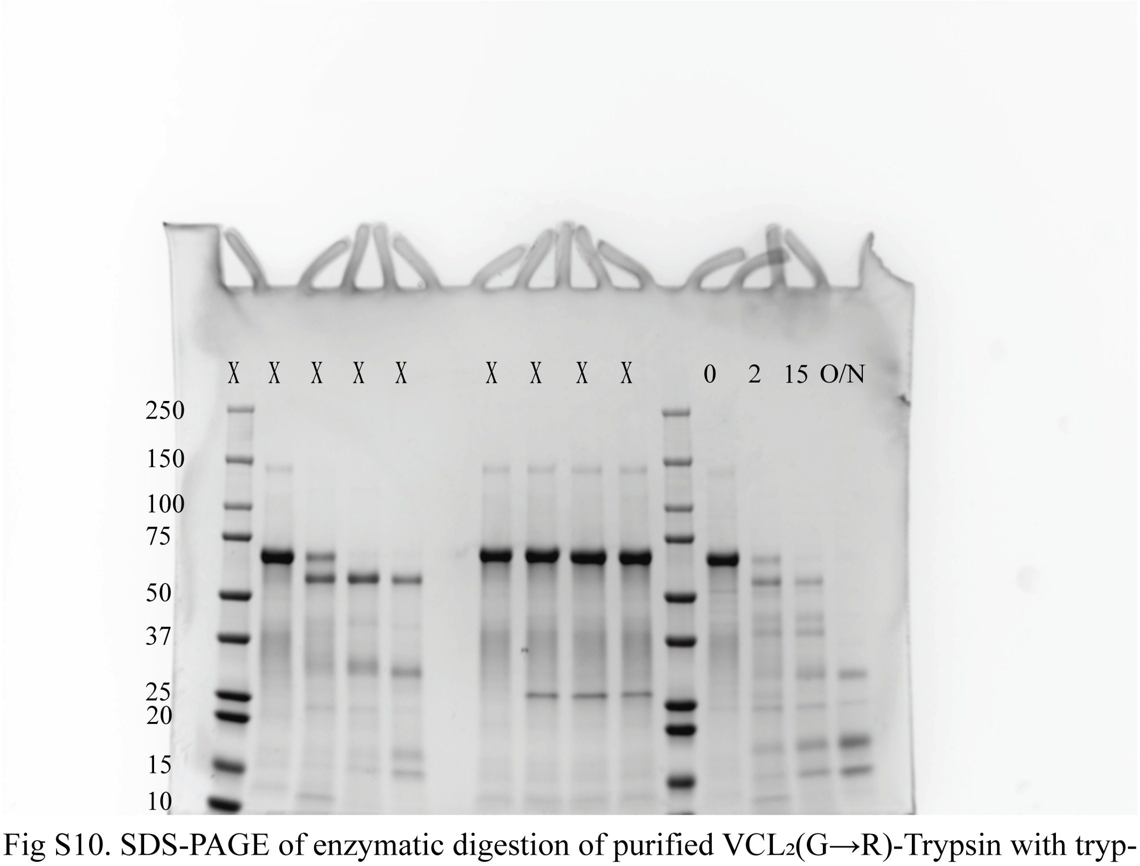

Fig S10. SDS-PAGE of enzymatic digestion of purified VCL_2(G→R)-Trypsin with trypsin for different time points (0 mins, 2 mins, 15 mins, and overnight (O/N)). Gels were stained with Coomassie blue, destained, and imaged using Gel Doc Imager. The gel image was used to generate Fig 5B in the manuscript. Molecular weight standards are in kDa.

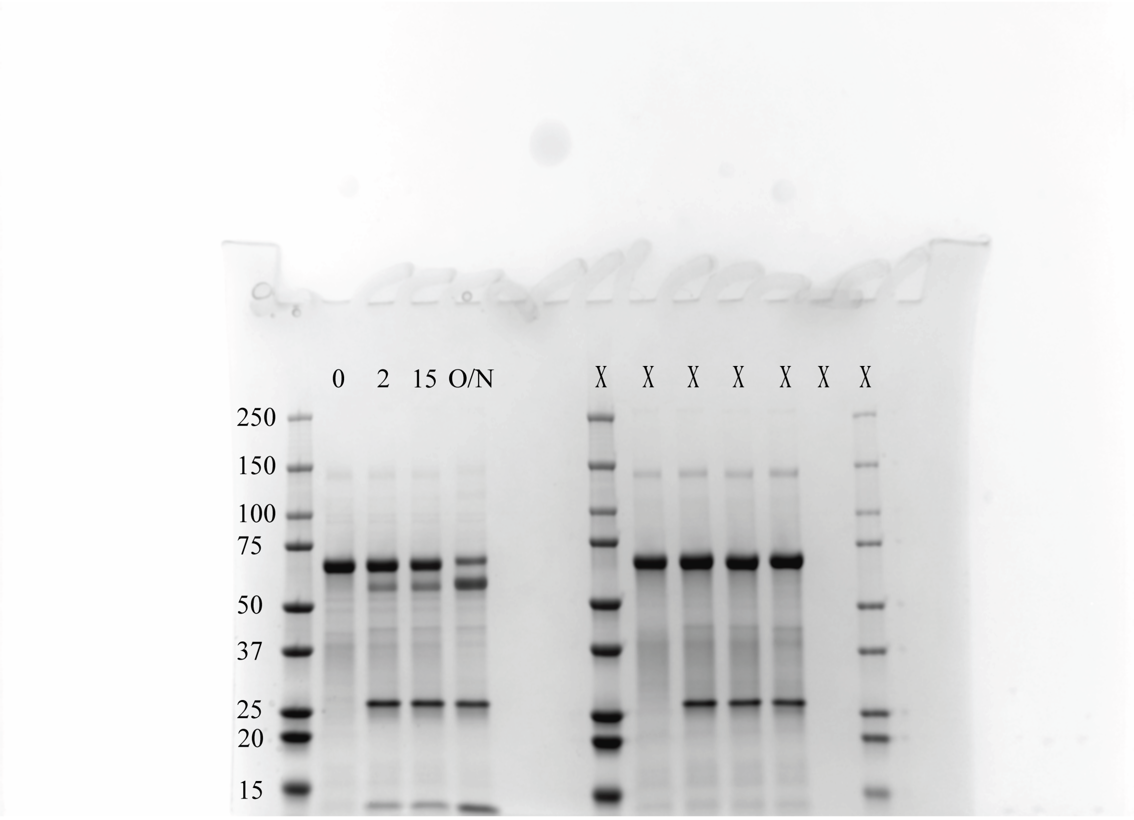

Fig S11. SDS-PAGE of enzymatic digestion of purified VCL_2-TEV with TEV protease for different time points (0 mins, 2 mins, 15 mins, and overnight (O/N)). Gels were stained with Coomassie blue, destained, and imaged using Gel Doc Imager. The gel image was used to generate Fig 5C in the manuscript. Molecular weight standards are in kDa.

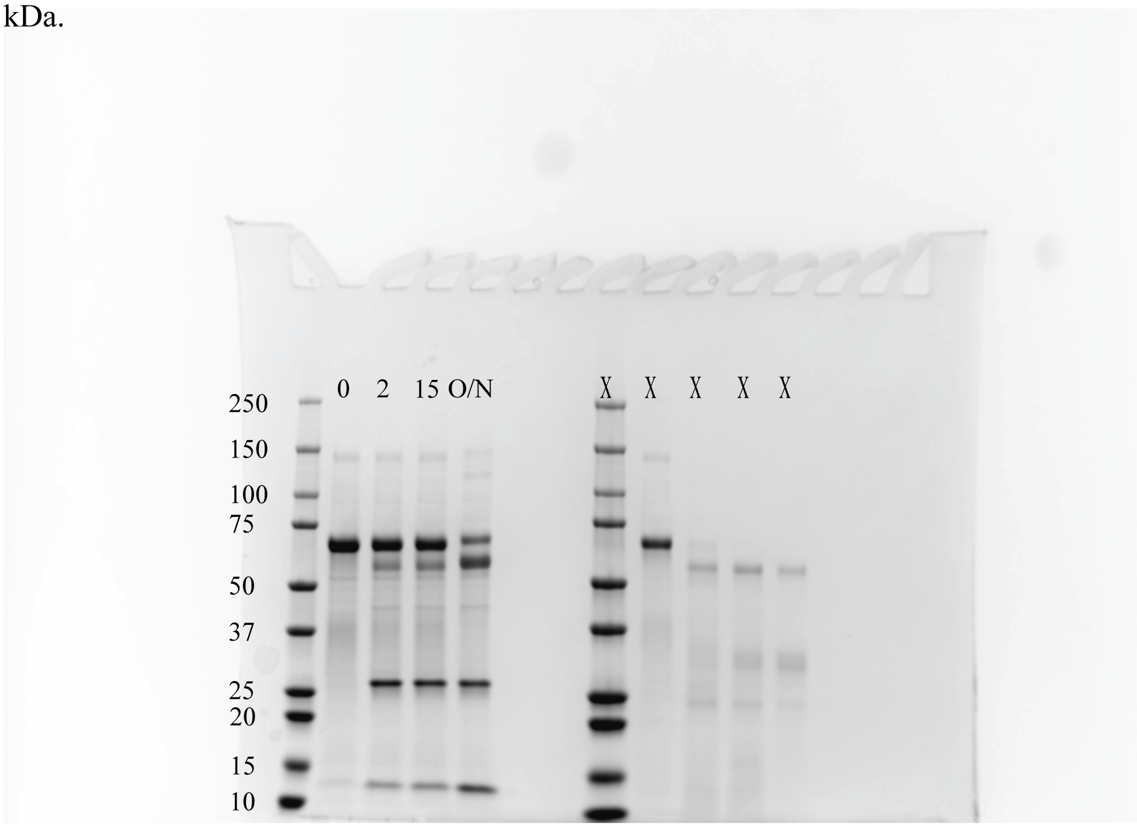

Fig S12. SDS-PAGE of enzymatic digestion of purified VCL_2(G→R)-TEV with TEV protease for different time points (0 mins, 2 mins, 15 mins, and overnight (O/N)). Gels were stained with Coomassie blue, destained, and imaged using Gel Doc Imager. The gel image was used to generate Fig 5D in the manuscript. Molecular weight standards are in kDa.

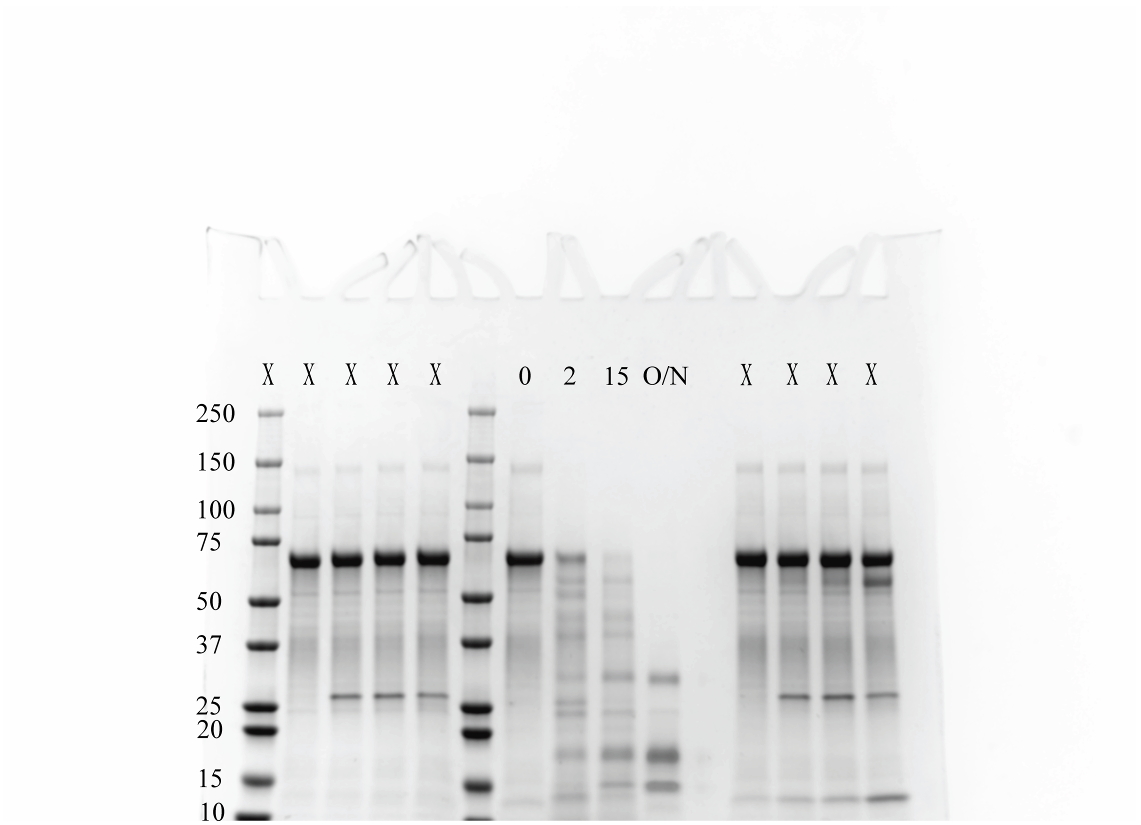

Fig S13. SDS-PAGE of enzymatic digestion of purified VCL_2(G→R)-TEV with trypsin for different time points (0 mins, 2 mins, 15 mins, and overnight (O/N)). Gels were stained with Coomassie blue, destained, and imaged using Gel Doc Imager. The gel image was used to generate Fig 5E in the manuscript. Molecular weight standards are in kDa.
