# Supplementary material for: Purification of recombinant bacterial collagens containing structural perturbations
Source: PLoS One. 2023 May 17;18(5):e0285864. doi: 10.1371/journal.pone.0285864 (PMC10191275; doi:10.1371/journal.pone.0285864)
Supplement: Attachment — Submitted filename: Response to Reviewers.docx [file pone.0285864.s002.docx]

PONE-D-23-06762

Purification of recombinant bacterial collagens containing structural perturbations

We would like to thank the reviewers for their effort and time in reviewing the manuscript. We sincerely appreciate their valuable and insightful comments, which helped us in improving the quality of the manuscript. We have addressed the comments specific to each reviewer below.

**Reviewer 1**

1. Specify the rationale for choosing to substitute Glycine with Arginine.

*Thank you for the suggestion. We have included the rationale for choosing Gly→Arg mutation in lines 83-87.*

*Lines 83-87:*

*Structurally distorted triple helices were generated through the introduction of Gly→Arg mutations within the integrin-binding site of CL domains. The Gly→Arg mutation would provide differential folding effects and has been associated with collagen disorders, including Osteogenesis Imperfecta and Ehlers-Danlos Syndrome and its subtypes (https://eds.gene.le.ac.uk/).*

2. Indicate the number of the amino acid substitution or specify which glycine was substituted in the integrin binding site.

*As listed in lines 120-122, Gly at position #201 and #444 were mutated to Arg residues. Within the integrin-binding site, GFP****G****ER, the second Gly residue (highlighted in bold) was mutated to Arg amino acid, which has further explained in lines 176-178 and Fig 2 legend.*

*Lines 176-178:*

*The VCL_2_(G→R) mutant protein includes a single Gly→Arg mutation (GFP****R****ER) near the middle of the CL domain within the IBS sequence to have minimum impact on the folding and assembly of the triple helix*

*Fig 2B:*

*Version of the VCL_2_ construct with Gly→Arg within the IBS, where the second Gly residue of GFPGER was mutated to Arg (highlighted by “★”).*

3. Reference for the contribution of the mutation Gly to Arg change the structure of CLP.

*Reference 22 (line 238) supports the impact of Gly→Arg mutation on the triple helix structure. Additional references have been added in support of the argument (28-30).*

4. Reference or argument that Gly to X would have same effect as Gly to Arg.

*We have added text in lines 252-256 justifying this claim.*

*Lines 252-256:*

*Replacing the trypsin cleavage site with TEV offers a reliable method to isolate CL domains containing structural perturbations irrespective of the type of substituted amino acid, the location of the mutation, and the surrounding local sequence, all of which play a decisive role in determining the conformation and stability of the triple helix.*

5. Figure 1:
   a. Panel C: Missing figure text.

*It is unclear which figure text is missing. The submitted Fig 2C contains a table, which describes the design of protein constructs, including the presence or absence of Gly substitution and the type of protease cleavage site (trypsin vs. TEV).*

b. Panel A:
   i. Exchange “Protease cleavage sites” with “TEV or Trypsin cleavage site”.
   ii. Specify which glycine was mutated in the integrin binding site

i. *We have exchanged the “protease cleavage sites” with “TEV or Trypsin cleavage site” in Fig 2A.*
ii. *We have updated the Fig 2B legend to highlight which Gly residue was mutated to Arg within the integrin-binding site.*

*Fig 2B:*

*Version of the VCL2 construct with Gly→Arg within the IBS, where the second Gly residue of GFPGER was mutated to Arg (highlighted by “★”).*

6. Line 79: forgo forgot

*There is no spelling error in line 79. Because of Gly→X mutations on the triple helix structure, it is challenging to remove an affinity tag and isolate collagen-like domains containing such mutations using trypsin enzyme. For that reason, authors intentionally do not cleave the affinity tag to prevent protein degradation.*

**Reviewer 2**

1. This manuscript by Gahlawat et al described a simple laboratory effort to purify a collagen like protein (CLP) having Gly-to-X mutations that was first expressed as a fusion protein. They provided evidence showing protease TEV is better suited for the removal of the His-tagged V-domain than trypsin because of the folding problem related to the mutation.
   a. Yet, there is no data and no mention on the folding of the peptides.

*We have added circular dichroism (CD) spectra in Supplementary Information (Fig S1) for the different CLPs demonstrating that the constructs properly fold into a triple helix as evidenced by a minimum and a maximum peak around 198-200 nm and 220-225 nm, respectively.*

Fig S1: Circular Dichroism (CD) spectra of purified proteins: VCL2-Trypsin, VCL2-TEV, VCL2(G→R)-Trypsin, and VCL2(G→R)-TEV.

b. Did the digestion act on monomer during purification or on folded trimer?

*The trypsin digestion assays were performed on the folded trimers and the digestion profile was visualized using SDS-PAGE. Due to the inclusion of denaturing chemicals and reducing agents, only the monomers will be seen.*

c. Can the triple helix form without the removal of V-domain? If not, how can they claim the varied sensitivity of the fusion protein to proteases is related to the ‘partially folded structure’?

*Yes, the bacterial collagen triple helix can form without the removal of V-domain.*

2. Figure 1 is missing.

*Fig 1 is attached.*

3. The optimization experiments are notoriously difficult to reproduce. The authors did not mention the reproducibility of the results

*We agree with the reviewer that optimization experiments are challenging. Using the reagents and chemicals listed in the Materials and Methods section of the manuscript, we obtained similar yields and purity of proteins in multiple batches, thus offering reproducible results. Since multiple factors can impact protein expression and purification, which ultimately affects protein yield and purity, it is always recommended to tailor the expression and purification of each protein construct by screening different culturing conditions. We emphasize that optimization is not the main theme of the paper.*
